# Recovery of Linear and Nonlinear Heart Rate Variability Metrics After Short‐Term Moderate versus Vigorous Intensity Exercise: A Cross‐Sectional Randomized Cross‐Over Study

**DOI:** 10.1002/ejsc.70077

**Published:** 2025-11-01

**Authors:** Thomas Gronwald, Hannes Kock, Lisa Röglin, Martin Möhle, Eva Kircher, Olaf Hoos, Sascha Ketelhut

**Affiliations:** ^1^ Institute of Interdisciplinary Exercise Science and Sports Medicine MSH Medical School Hamburg Hamburg Germany; ^2^ G‐Lab Faculty of Applied Sport Sciences and Personality BSP Business and Law School Berlin Germany; ^3^ Department of Health Sciences Swedish Winter Sports Research Centre Mid Sweden University Östersund Sweden; ^4^ Department of Endurance Science Institute for Applied Training Science Leipzig Germany; ^5^ Institute of Sport Science Martin Luther University Halle‐Wittenberg Halle Germany; ^6^ Department of Medical Pedagogy SRH Berlin University of Applied Sciences Berlin Germany; ^7^ Faculty of Human Sciences Center for Sports and Physical Education Julius‐Maximilians‐University Wuerzburg Wuerzburg Germany; ^8^ Institute of Sport Science University of Bern Bern Switzerland

**Keywords:** autonomic nervous system, DFAa1, endurance sports, exercise intensity, HRV, parasympathetic reactivation

## Abstract

The present study explored acute responses of heart rate (HR) variability (HRV) metrics, incorporating the nonlinear index alpha 1 of detrended fluctuation analysis (DFAa1) during passive recovery, providing information about correlation properties of HR time series during the regulation of recovery processes. Recreationally active female (*n* = 13) and male (*n* = 13) participants participated. In a first session, a graded exercise test was conducted to determine peak HR (HR_PEAK_) and peak oxygen consumption (VO_2PEAK_). In a second and third session, participants completed an endurance training with moderate intensity (MOD) on a treadmill and an exergaming training with vigorous intensity (VIG), randomized and counterbalanced. Before and up to 45 min after the respective exercise sessions, RR‐interval and hemodynamic measurements (peripheral systolic, SBP; diastolic blood pressure, DBP; and pulse wave velocity, PWV) were conducted. Internal load analysis of MOD versus VIG revealed significant differences and appropriate prescription of intensity domains during exercise (%HR_PEAK_: ∼66% vs. 86% and %VO_2PEAK_: ∼48% vs. 66%). The present data showed significant main effects of time, intensity, and their interaction for all RR‐interval outcomes, PWV, and SBP. DFAa1 demonstrated a stronger correlated reorganization and overcompensation after VIG, with higher values and therefore increased correlation properties throughout the recovery process. The present data suggest that VIG transiently delays the recovery of cardiac parasympathetic activity and the normalization of correlation properties of HR time series. Regarding acute early and delayed recovery processes, higher correlation properties may reflect more order (less complexity) and interaction of involved physiological subsystems, supporting the assumption of increased systemic control to process the demands of higher exercise intensity.

## Highlights

- Higher exercise intensity results in delayed reorganization, transiently delays parasympathetic reactivation, and decreases values of linear HRV metrics during passive recovery.
- The present study adds to the current body of research by showing that DFAa1, as a nonlinear metric of HRV, exhibits a stronger correlated reorganization and overcompensation after a vigorous compared to a moderate exercise bout.
- Higher correlation properties may indicate more order and interaction of the involved control processes managing recovery and suggests a stronger systemic control to process the demands of higher exercise intensities.
- Further research is needed to verify the potential of using standardized postexercise linear and nonlinear HRV metrics as a monitoring tool for individual cardiac reactivation patterns.

## 1 Introduction

Appropriate exercise and training prescription in endurance sports faces the challenge of balancing individualized organismic stimuli with recovery phases. This balance is essential to trigger homeostatic perturbations and to initiate desirable metabolic, cardiovascular, and/or neuromuscular adaptations (Mujika 2017; Borresen and Lambert 2009). For this purpose, general training principles provide guidance on appropriate exercise intensity as well as volume distribution within training schedules (Lambert et al. 2008; Issurin 2010; Kasper 2019). Although high volumes of low‐intensity exercise are essential for the long‐term development of endurance capacity, the incorporation of high‐intensity exercise seems crucial to provide an appropriate stimulus and to reach specific adaptations (Mølmen et al. 2024; Seiler 2024). However, scheduling such stimuli at the right time within an appropriate homeostatic state of enhanced performance and recovery level is essential (Bompa and Buzzichelli 2019). Without sensitive monitoring of acute and chronic recovery, training adaptations and performance levels could be adversely affected.

Recovery is the process of restoring capacity to a previous or improved level and involves the integrated response of various physiological subsystems. These subsystems contribute to homeodynamic regulation and processing of exercise stimuli acutely and chronically (Stanley et al. 2013). Inappropriate intensity distribution in the short‐term could lead to glycogen depletion (Beneke et al. 2011), gastrointestinal barrier disruption (van Wijck et al. 2012), overall central and muscular fatigue (Noakes et al. 2005; Venhorst et al. 2018), and delayed cardiac parasympathetic reactivation, a marker of cardiovascular or autonomic recovery (Stanley et al. 2013). To date, monitoring of resting conditions alongside metrics of heart rate (HR) time series has a broad state of research (Achten and Jeukendrup 2003; Aubert et al. 2003; Bellenger et al. 2016; Michael et al. 2017; Lundstrom et al. 2023; Gronwald et al. 2024a). The goal is to gather sensitive information about recovery and the status of the autonomic nervous system (ANS) at the interface of the central autonomic network (CAN, Benarroch 1993) and its multilayered feedforward and feedback loops. In this regard, the assessment of cardiac parasympathetic reactivation was introduced to analyze HR variability (HRV) metrics following exercise sessions, with the aim of evaluating acute responses and physiological perturbations due to prescribed intensity and volume of the respective exercise sessions (Stanley et al. 2013).

HRV describes the beat‐to‐beat variability of the HR over a defined measurement period and reflects the dynamic end‐organ response of the heart to physiologic and/or pathologic perturbations (Billman et al. 2015). This complex modulation integrates several feedforward and feedback mechanisms that act on different time scales and is therefore linked to the dynamic regulation of circulatory control of arterial blood pressure and HR to meet the context‐dependent needs of the entire organism (Gronwald, Schaffarczyk, et al. 2024). Specific HRV metrics from the time‐, frequency‐, and/or nonlinear domains capture quite different HRV components, each more or less associated with physiological processes. Their interpretation should be context‐sensitive and dependent on the applied setting (Task Force, 1996; Sassi et al. 2015; Shaffer and Ginsberg 2017). Specific metrics of HRV primarily reflect vagal modulation, providing insights for fatigue, or organismic readiness monitoring (J. J. Goldberger et al. 2006; Plews et al. 2013; Buchheit 2014).

Postexercise analysis of HRV metrics was introduced with the belief of providing more sensitive information about training response and adaptation compared to resting HRV or HR recovery analysis (Yamamoto et al. 2001; Buchheit 2014). However, acute responses are highly individual, and the underlying physiological mechanisms are not fully understood (Stanley et al. 2013; Michael et al. 2017). Previous studies have demonstrated specific patterns in postexercise recovery cardiac parasympathetic reactivation across different populations and in response to different characteristics of aerobic exercise (Stanley et al. 2013; Michael et al. 2017; Price et al. 2020; Leal‐Menezes et al. 2025). Faster parasympathetic reactivation and restoration of autonomic HR control have been observed after low‐intensity exercise bouts compared to exercise bouts of higher intensities (Michael et al. 2017). Moreover, a higher performance level is associated with faster reorganization (Seiler et al. 2007). Key determinants of postexercise HRV include the sudden removal of “central command”, abolished feedback from muscle mechanoreceptors (during passive recovery), blood pressure regulation, activity state, and degree of stimulation of baroreflex and metaboreflex, along with sympathetic withdrawal and the reactivation of the parasympathetic branch of the ANS (Buchheit et al. 2007; Stanley et al. 2013; Michael et al. 2017). In addition, methodological considerations of protocol variation should be acknowledged (e.g., active vs. passive recovery and recovery posture; Michael et al. 2017).

Methodological approaches for assessing postexercise cardiac autonomic recovery have been developed (Peçanha et al. 2017), highlighting the implementation of nonlinear metrics of HR time series as crucial to analyze the complex cardiac autonomic activity during postexercise recovery (Medeiros et al. 2017). Nonlinear metrics complement linear metrics of HRV and rather focus on qualitative characteristics, (fractal) dynamics, and complexity of the time series (de Godoy 2016). Nonlinear methods, such as the detrended fluctuation analysis and its short‐term scaling exponent alpha1 (DFAa1), have previously demonstrated good reliability during active recovery (Boullosa et al. 2014). Analyses of DFAa1 during acute endurance exercise indicated that this metric may be a sensitive marker for assessing global organismic demands (Gronwald and Hoos 2020; Gronwald et al. 2020). It has also been shown to be useful as a marker of acute fatigue (Rogers et al. 2021; Schaffarczyk et al. 2022a; Van Hooren, Bongers, et al. 2023; b) and as a potential internal load measure of durability in studies involving prolonged exercise (Gronwald et al. 2024; Nuuttila et al. 2024; Rogers et al. 2025). Therefore, expanding these findings to future approaches of recovery assessment seems promising. Although there are several studies assessing postexercise HRV (for overview see Michael et al. 2017), there are only a few studies analyzing nonlinear HRV metrics in general and DFAa1 in specific during active and passive recovery (Casties et al. 2006; Platisa and Gal 2008; Mendonca et al. 2010; Blasco‐Lafarga et al. 2013; Boullosa et al. 2014; Gronwald et al. 2019; Martínez‐Navarro et al. 2019). In addition, comparisons of the effects of different exercise stimuli (e.g., intensity) on nonlinear postexercise HRV are scarce (Millar et al. 2009; Perkins et al. 2017; Naranjo‐Orellana et al. 2020).

Therefore, the aim of the present study was to expand the state of research by determining the acute responses of HR time‐series metrics and selected hemodynamic parameters during the recovery phase after two exercise bouts with different intensities. The main focus was to replicate the results of previous studies on linear metrics of HRV analysis and to advance the research by incorporating the nonlinear metric DFAa1, providing additional qualitative information about correlation properties of HR time series during recovery. We hypothesized that the more intense exercise bout would initiate greater perturbations during recovery, leading to delayed reorganization and decreased values of linear HRV metrics. Additionally, we hypothesized that DFAa1 would show a stronger correlated reorganization and overcompensation with higher values following the more intense exercise bout.

## 2 Methods and Materials

### 2.1 Participants

A total of 26 recreationally active female (*n* = 13) and male (*n* = 13) participants (age: 24.8 ± 4.0 years, body mass: 68.3 ± 10.8 kg, height: 171.2 ± 9.6 cm, and habitual physical activity: 7.3 ± 3.5 h/week) volunteered to participate in the study. The inclusion criteria for female participants required a regular and healthy menstrual cycle and no reported history of menstrual distress for the past 6 months. Overall, exclusion criteria were an underlying health condition, orthopedic injuries, and regular use of antihypertensive or other cardiovascular medications. All participants were informed about the risks and benefits of the procedures and signed a written informed‐consent form. The ethics committee of the Medical Faculty of the Martin‐Luther‐University Halle‐Wittenberg (reference no.: 2019–177) approved the experimental protocol of this cross‐sectional study. The study was carried out in accordance with the principles set forth in the most recent revision of the Declaration of Helsinki.

### 2.2 Study Design

This study is based on data from a cross‐sectional study preregistered at ISRCTN registry (ISRCTN43067716, 38154), which aimed to investigate the exercise stimuli of an exergaming session and the subsequent cardiovascular reactivity thereafter (Ketelhut et al. 2022). Participants took part in three experimental sessions. In each session, they were instructed to visit the laboratory 4 hours postprandial and to refrain from consuming caffeinated or alcoholic beverages and nicotine during this period. The participants were further advised to avoid intensive physical exercise for at least 12 h before the sessions. All tests were conducted at similar times of the day, with a 48‐h washout period between consecutive visits and under standardized conditions in the same laboratory with a room temperature of 23°C. For female participants, the examination days were selected so that they did not fall into the early follicular phase, where the postexercise hypotension effect is supposed to be more pronounced (Esformes et al. 2006). In the first session, demographic and anthropometric data were obtained. Furthermore, a graded exercise test was conducted. In the second and third session, participants completed an endurance training with moderate intensity (MOD) on a treadmill or an exergaming training with vigorous intensity (VIG, upper range of the “high” intensity domain, Bishop et al. 2025) in a randomized and counterbalanced order. RR‐interval and hemodynamic measurements were conducted before and during 45 min of passive recovery in a supine position following the respective exercise sessions.

**FIGURE 1:**
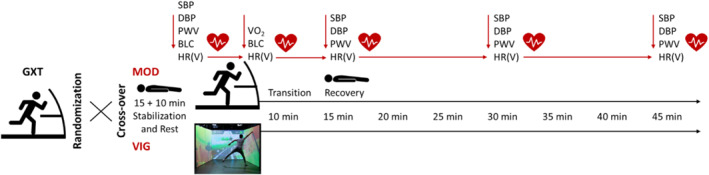
Overview of the study design. BLC, blood lactate concentration; DBP, peripheral diastolic blood pressure; GXT, graded exercise test; HR(V), heart rate (variability); MOD, moderate‐intensity continuous treadmill running exercise; PWV, pulse wave velocity; SBP, peripheral systolic blood pressure; VIG, vigorous‐intensity intermittent exergaming; VO_2_, oxygen consumption.

In addition, RR‐intervals were recorded during the exercise sessions, and blood lactate concentrations (BLC) were measured before and immediately at the end of the exercise bouts (see study design in Figure 1).

### 2.3 Test Procedures

During the first session, participants completed baseline surveys assessing demographics, habitual physical activity, and medical history. Height and body mass were measured with a stadiometer and a scale (BC‐545 Innerscan, Tanita, Netherlands). Furthermore, a blood pressure measurement was performed to familiarize participants with the procedure. The participants then completed a graded exercise step test on a treadmill (h/p/cosmos, Pulsar 3p, Nussdorf‐Traunstein, Germany) until voluntary exhaustion (start: 7.5–10.5 km/h depending on individual training status, duration: 3 min, increment: 1.5 km/h, and pause: 1 min passive rest to collect BLC samples from the earlobe) to determine peak HR (HR_PEAK_) and peak oxygen consumption (VO_2PEAK_). Afterward, participants were familiarized with the exergaming condition and had the opportunity to test a tutorial‐simulation.

The MOD condition consisted of 35 min moderate endurance exercise on the same treadmill as for the graded exercise test. After 5 min of warm‐up at 5.5 km/h, the treadmill speed was set according to the individual aerobic threshold (based on BLC beginning to rise above baseline level) and continuously adjusted until the participants reached a HR of approx. 65%–70% of their HR_PEAK_ to target a moderate intensity domain (Garber et al. 2011; Bishop et al. 2025). For the VIG condition, the exergame setup ExerCube (Sphery AG, Au, Switzerland, see Figure 1) was used (Martin‐Niedecken et al. 2020), allowing the player to engage in an individually tailored, whole‐body, and functional exercise session. The game setup consists of three walls that surround the player, which serve as projection screens and a haptic interface for bodily interactions, allowing an immersive gaming experience. The player wears two HTC Vive‐trackers on their wrists and two on their ankles, which continuously track the player's movements and body position. In the present study, participants played the game Sphery Racer in a single‐player mode during the exercise session (Martin‐Niedecken et al. 2020). In this game, the player navigates an avatar on a hoverboard along a racing track and performs different movement tasks (e.g., squats, launches, punches, and burpees). Overall, the game scenario lasted about 27 min and implemented five dynamic movement levels (level 1: 2.5 min; level 2: 2.5 min; level 3: 5 min; level 4: 5 min; and level 5: 10 min), with exercise intensity gradually increasing in each level. The levels are interspersed by short recovery periods of approx. 30 s. Throughout the game, the difficulty and complexity of the challenges are adjusted based on the player's cognitive skills. If the player makes too many mistakes, the speed and complexity of the game decrease—if the player makes no mistakes, both parameters increase gradually. This allows an individually tailored training stimulus. It was intended that the mean exercise intensity of the VIG condition would be in the vigorous domain (Garber et al. 2011; upper range of the “high” intensity domain according to Bishop et al. 2025). Further information about the game characteristics can be found in Ketelhut et al. (2022).

### 2.4 Data Recording

HR data were monitored continuously throughout the graded exercise step test as well as before, during, and after MOD and VIG, using a HR chest strap monitor (WearLink W.I.N.D. and RS800 CX, Polar Electro OY, Kempele, Finland; Hernando et al. 2018). RR‐interval data were only used for HRV analysis before and after MOD and VIG conditions. BLC was assessed after each stage of the graded exercise test as well as before and after MOD and VIG, using an enzymatic‐amperometric analysis method (Dr. Müller Gerätebau GmbH, Super GL ambulance, Freital, Germany). The collected data were processed utilizing the software WinLactat 3.1 (mesics GmbH, Münster, Germany), and individual BLC thresholds were derived from the lactate‐velocity curve using the Dickhuth model (Dickhuth et al. 1991). Oxygen consumption (VO_2_) was also recorded continuously (breath‐by‐breath) during the graded exercise test to assess VO_2PEAK_ as well as during MOD (excluding the warm‐up period) and VIG (excluding the initial 5 minutes of fast response kinetic) for internal load analysis. Here, a portable indirect calorimetric gas‐exchange analysis system MetaMax 3B (Cortex Biophysik GmbH, Leipzig, Germany) was used to allow unrestricted movement. The values obtained were averaged over 30 s (Nolte et al. 2023). In addition, before (PRE) and after (15, 30, and 45 min as POST15, POST30, and POST45) MOD and VIG, resting peripheral systolic (SBP in mmHg) and diastolic blood pressure (DBP in mmHg) as well as pulse wave velocity (PWV in m/s) were measured using the Mobil‐O‐Graph (24 PWA monitor, IEM, Stolberg, Germany) as a clinically validated device for hemodynamic measurements (Franssen and Imholz 2010). The measurement device operates with a novel transfer function‐like algorithm, using brachial cuff‐based waveform recordings. All resting measurements were obtained in a supine position; resting measurements before the exercise sessions were recorded after a 15‐min stabilization phase. A minimum of two readings were taken from the right upper arm using custom‐fit blood pressure cuffs performed by the same qualified staff member.

### 2.5 RR‐Interval Analysis

To analyze RR‐intervals and HR (in beats per minute, bpm), data were exported from the device and processed in Kubios HRV Premium Version 3.5.0 (Biosignal Analysis and Medical Imaging Group, Department of Physics, University of Kuopio, Kuopio, Finland). Preprocessing settings were set to the default values, including the RR detrending method, which was kept at “smoothness priors” (Lambda = 500). The RR‐interval series were then corrected using the Kubios HRV “automatic correction” method. Time‐domain metrics—mean RR‐intervals (meanRR in ms), the standard deviation of all normal‐to‐normal RR‐intervals over a given time interval (SDNN in ms), and root mean square of successive differences of normal‐to‐normal RR‐intervals (RMSSD in ms)—were analyzed. DFAa1 was calculated (Peng et al. 1995) with a window size of 4 ≤ *n* ≤ 16 beats. Data were also scanned visually for artifacts (e.g., spikes in RR‐interval series which were not corrected by the artifact correction algorithm), marked as “noise” and removed manually. Datasets with > 3% artifacts were excluded from HRV analysis. All resting measurement metrics were analyzed over a time segment of 3 minutes (resting condition before the exercise sessions: 06:00–09:00 min of a 10‐min recording as PRE; recovery condition after the exercise sessions: 01:30–04:30 min of a 5‐min recording interval every 5 minutes for 35 min, after an acute 10‐min transition recovery phase as POST15, POST20, POST25, POST30, POST35, POST40, and POST45). Mean and maximum HR values during MOD (excluding the warm‐up period) and VIG (excluding the initial 5 minutes of fast response kinetic) were analyzed over the course of the exercise bouts.

### 2.6 Statistical Analysis

**FIGURE 2:**
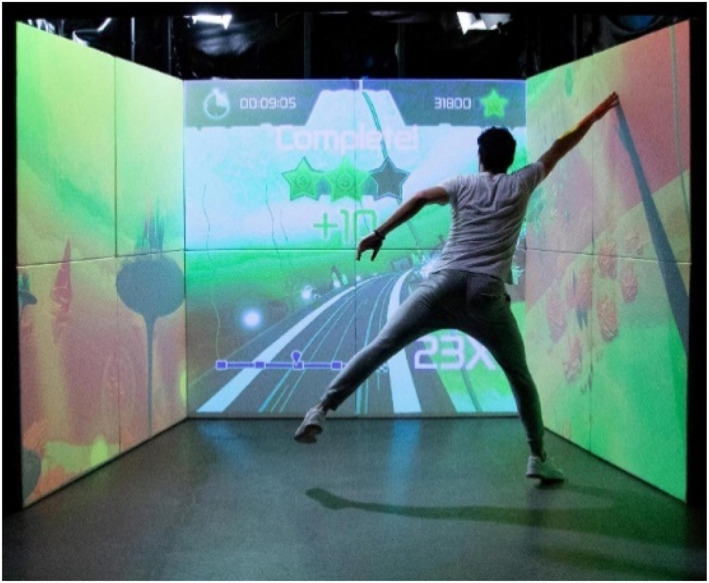
One participant in the ExerCube (see description in section “test procedures”).

All statistical analysis were performed using jamovi version 2.5 (https://www.jamovi.org) and Microsoft Excel (Microsoft Corp, Redmond, USA). Data are presented as mean ± standard deviation (SD). Normality was assessed using the Shapiro–Wilk test and visual inspection of QQ plots. Individual HR_PEAK_ and VO_2PEAK_, measured during the graded exercise step test, were used to calculate the percentage of HR_PEAK_ (%HR_PEAK_) and VO_2PEAK_ (%VO_2PEAK_) achieved during the exercise sessions for each participant. Linear mixed models incorporating within‐subject and between‐intensity factors (MOD and VIG) were used to examine the effect of time on all variables (PWV, SBP, DBP, HR, meanRR, SDNN, RMSSD, and DFAa1), with time and condition as a fixed effect and subject ID as random effect with a random intercept to account for individual variability. To evaluate sex differences, sex was added as an additional factor to each model. Models were fitted using restricted maximum likelihood (REML). Post hoc testing was performed using Bonferroni correction. For the analysis, the absolute changes of dependent variables were calculated in reference to the respective resting values (e.g., POST15‐PRE). In addition, paired *t*‐tests were applied to analyze internal load differences between the conditions (mean HR, HR_PEAK_, %HR_PEAK_, mean VO_2_, VO_2PEAK_, %VO_2PEAK_, and BLC). Statistical tests were deemed to be significant at *p* ≤ 0.05 (Figure 2).

## 3 Results

All participants completed the three experimental sessions without adverse events. The participants reached a HR_PEAK_ of 193.5 ± 7.6 bpm (MIN: 176 bpm and MAX: 207 bpm), a VO_2PEAK_ of 48.6 ± 5.6 mL/min/kg (MIN: 39 mL/min/kg and MAX: 62 mL/min/kg), and a BLC of 9.1 ± 1.9 mmol/L (MIN: 5.1 mmol/L and MAX: 12.5 mmol/L) at voluntary exhaustion during the treadmill step test. Due to artifact correction, we had to exclude 22% of RR‐interval data for the MOD and 23% for the VIG condition.

During MOD, the participants reached a mean HR of 127.5 ± 8.2 bpm (% HR_PEAK_: 66.1 ± 3.7%) and a maximum HR of 151.2 ± 10.4 bpm (%HR_PEAK_: 78.5 ± 4.0%). Mean VO_2_ was 24.1 ± 2.7 mL/min/kg (% VO_2PEAK_: 47.8 ± 10.8%), with a maximum VO_2_ of 31.1 ± 3.9 mL/min/kg (%VO_2PEAK_: 64.5 ± 5.6%). During VIG, mean HR (166.7 ± 11.1 bpm and %HR_PEAK_: 86.1 ± 4.3%; *p* < 0.001) and maximum HR (187.0 ± 9.4 bpm and %HR_PEAK_: 96.7 ± 3.6%; *p* < 0.001) were significantly higher. Mean VO_2_ (32.0 ± 3.8 mL/min/kg and % VO_2PEAK_: 66.1 ± 5.8%; *p* < 0.001) and maximum VO_2_ (41.1 ± 4.8 mL/min/kg and % VO_2PEAK_: 84.9 ± 7.8%; *p* < 0.001) were also significantly higher during VIG. Furthermore, BLC values were significantly higher immediately after the VIG bout compared to MOD (4.8 ± 3.0 vs. 1.0 ± 0.3 mmol/L; *p* < 0.001), whereas no differences could be detected during the resting condition (PRE) prior exercise (0.8 ± 0.3 vs. 0.8 ± 0.2 mmol/L; *p* = 0.822).

The comparisons of resting conditions before and during recovery following MOD and VIG revealed main effects of time, intensity, and the interaction for PWV and SBP. For DBP, only a significant main effect of time was found. Additionally, no effects of sex were observed. Furthermore, we found significant main effects of time, intensity, and the interaction for all RR‐interval outcomes (see Table 1).

**TABLE 1:** Summary of main effects for time, condition (intensity), and interaction for hemodynamic and RR‐interval outcomes.

Parameters	Time	Intensity	Interaction
PWV [m/s]	*F* = 5.34 and *p* = 0.002	*F* = 27.40 and *p* < 0.001	*F* = 5.12 and *p* = 0.002
SBP [mmHg]	*F* = 15.12 and *p* < 0.001	*F* = 23.46 and *p* < 0.001	*F* = 5.44 and *p* = 0.001
DBP [mmHg]	*F* = 11.09 and *p* < 0.001	*F* = 1.90 and *p* = 0.169	*F* = 1.28 and *p* = 0.283
HR [bpm]	*F* = 62.14 and *p* < 0.001	*F* = 368.82 and *p* < 0.001	*F* = 12.12 and *p* < 0.001
meanRR [ms]	*F* = 44.98 and *p* < 0.001	*F* = 244.63 and *p* < 0.001	*F* = 6.41 and *p* < 0.001
SDNN [ms]	*F* = 8.36 and *p* < 0.001	*F* = 65.80 and *p* < 0.001	*F* = 2.17 and *p* = 0.037
RMSSD [ms]	*F* = 9.41 and *p* < 0.001	*F* = 84.68 and *p* < 0.001	*F* = 2.38 and *p* = 0.022
DFAa1	*F* = 11.72 and *p* < 0.001	*F* = 68.02 and *p* < 0.001	*F* = 2.67 and *p* = 0.011

Abbreviations: DBP, peripheral diastolic blood pressure; DFAa1, short‐term scaling exponent alpha 1 of detrended fluctuation analysis; HR, heart rate; meanRR, mean RR‐intervals; PWV, pulse wave velocity; RMSSD, root mean square of successive differences of normal‐to‐normal RR‐intervals; SBP, peripheral systolic blood pressure; SDNN, standard deviation of all normal‐to‐normal RR‐intervals.

When comparing the different measurement time points for MOD, a significant increase in HR (*p* < 0.001) could be detected between PRE versus POST15. Additionally, between POST15 and POST45, a significant decrease in HR (*p* < 0.001) could be shown. No changes were observed in hemodynamic parameters or the other RR‐interval metrics. For VIG, no significant changes in hemodynamic parameters were observed between PRE and POST15. However, at POST45, the values were significantly lower compared to PRE (PWV: *p* = 0.007, SBP: *p* < 0.001, and DBP: *p* < 0.001) and POST15 (all *p* < 0.001). Regarding RR‐interval metrics, meanRR, SDNN, and RMSSD showed significantly decreased values (all *p* < 0.001), whereas HR and DFAa1 were significantly increased (both *p* < 0.001) at POST15 compared to PRE. SDNN (*p* = 0.005) and RMSSD (*p* = 0.023) significantly increased from POST15 to POST45, whereas HR significantly decreased (*p* < 0.001). The comparison between POST45 and PRE revealed a significantly higher HR (*p* < 0.001) and DFAa1 (*p* = 0.007).

When comparing MOD and VIG, PWV showed significantly lower values at POST45 following VIG (*p* < 0.001), and SBP was significantly lower at POST30 (*p* = 0.004) and POST45 (*p* < 0.001) after VIG. For the RR‐interval metrics, SDNN (*p* = 0.042) and RMSSD (*p* = 0.002) were significantly lower at POST15 after VIG, whereas HR (*p* < 0.001) and DFAa1 (*p* < 0.001) were significantly higher. HR remained significantly higher (*p* < 0.001) up to POST45 after VIG. For SDNN (*p* = 0.035) and RMSSD (*p* = 0.005), significantly lower values were observed until POST30 after VIG.

**FIGURE 3:**
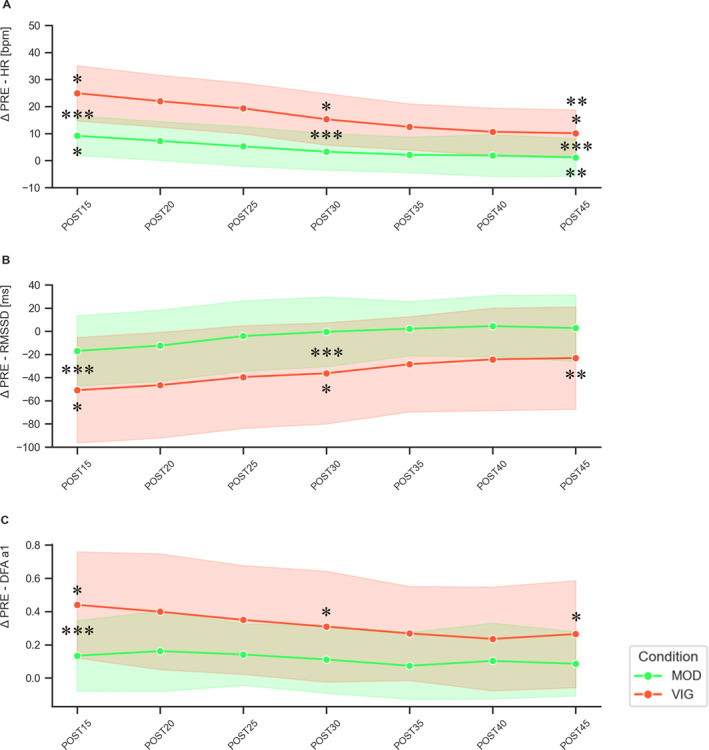
Absolute changes (±SD) of HR (A), RMSSD (B), and DFAa1 (C) from resting conditions before (ΔPRE) to specific time points 15–45 min following (POST15‐POST45) moderate‐intensity continuous treadmill running exercise (MOD) and the vigorous‐intensity intermittent exergaming (VIG). *significant compared to PRE, **significant compared to POST15, and ***significant compared to MOD.

All hemodynamic and RR‐interval resting and recovery data for MOD and VIG are displayed in Table 2. Figure 3 shows absolute changes of HR, RMSSD, and DFAa1 over the course of the measurement time points.

**TABLE 2:** Summary of hemodynamic and RR‐interval outcomes (mean ± SD) during resting conditions before (PRE) and at specific time points 15–45 min following (POST15‐POST45) the moderate‐intensity continuous treadmill running exercise (MOD) and the vigorous‐intensity intermittent exergaming (VIG).

Parameters	PRE	POST15	POST20	POST25	POST30	POST35	POST40	POST45
MOD								
PWV [m/s]	5.11 ± 0.38	5.28 ± 0.44 (*p* = 0.115*)	—	—	5.17 ± 0.44 (*p* = 1.000*)	—	—	5.21 ± 0.44 (*p* = 1.000* and *p* = 1.000**)
SBP [mmHg]	118.2 ± 8.9	118.2 ± 11.6 (*p* = 1.000*)	—	—	116.8 ± 9.4 (*p* = 1.000*)	—	—	116.3 ± 9.6 (*p* = 1.000* and *p* = 1.000**)
DBP [mmHg]	68.9 ± 6.3	68.0 ± 8.1 (*p* = 1.000*)	—	—	66.5 ± 7.6 (*p* = 1.000*)	—	—	65.8 ± 6.1 (*p* = 0.216* and *p* = 1.000**)
HR [bpm]	60.6 ± 10.8	70.6 ± 11.5 (*p* < 0.001*)	69.3 ± 9.9	65.4 ± 11.3	65.4 ± 11.2 (*p* = 0.801*)	64.0 ± 10.7	62.6 ± 11.1	64.9 ± 11.2 (*p* = 1.000* and *p* < 0.001**)
meanRR [ms]	1018.9 ± 170.9	871.1 ± 138.9 (*p* < 0.001*)	881.1 ± 116.0	941.8 ± 154.3	942.6 ± 153.6 (*p* = 0.247*)	959.5 ± 149.6	958.5 ± 171.2	947.9 ± 152.2 (*p* = 1.000* and *p* < 0.001**)
SDNN [ms]	52.4 ± 21.8	40.2 ± 17.2 (*p* = 1.000*)	46.3 ± 20.2	52.8 ± 19.1	55.2 ± 20.5 (*p* = 1.000*)	56.5 ± 18.4	60.0 ± 18.0	52.3 ± 22.0 (*p* = 1.000* and *p* = 1.000**)
RMSSD [ms]	61.2 ± 27.5	41.9 ± 24.1 (*p* = 0.414*)	46.7 ± 23.9	56.9 ± 26.0	66.9 ± 31.2 (*p* = 1.000*)	63.2 ± 27.8	67.2 ± 29.3	57.5 ± 31.3 (*p* = 1.000* and *p* = 1.000**)
DFAa1	0.76 ± 0.25	0.91 ± 0.26 (*p* = 0.649*)	0.93 ± 0.24	0.90 ± 0.24	0.85 ± 0.26 (*p* = 1.000*)	0.81 ± 0.26	0.83 ± 0.25	0.84 ± 0.30 (*p* = 1.000* and *p* = 1.000**)
VIG								
PWV [m/s]	5.23 ± 0.44	5.26 ± 0.45 (*p* = 1.000*) and (*p* = 0.573***)	—	—	5.14 ± 0.45 (*p* = 1.000*) and (*p* = 0.276***)	—	—	5.02 ± 0.44 (*p* = 0.007*, *p* < 0.001**, and *p* < 0.001***)
SBP [mmHg]	120.8 ± 10.1	119.7 ± 14.4 (*p* = 1.000*) and (*p* = 1.000***)	—	—	114.7 ± 10.4 (*p* < 0.001*) and (*p* = 0.004***)	—	—	112.9 ± 10.0 (*p* < 0.001*, *p* < 0.001**, and *p* < 0.001***)
DBP [mmHg]	69.8 ± 6.9	68.5 ± 8.6 (*p* = 1.000*) and (*p* = 1.000***)	—	—	67.5 ± 9.3 (*p* = 1.000*) and (*p* = 1.000***)	—	—	64.0 ± 7.1 (*p* < 0.001*, *p* = 0.004**, and *p* = 0.542***)
HR [bpm]	62.3 ± 11.6	86.7 ± 14.8 (*p* < 0.001*) and (*p* < 0.001***)	85.3 ± 12.2	82.0 ± 12.9	76.7 ± 14.2 (*p* < 0.001*) and (*p* < 0.001***)	74.7 ± 13.6	73.6 ± 13.3	70.5 ± 11.9 (*p* < 0.001*, *p* < 0.001**, and *p* < 0.001***)
meanRR [ms]	995.9 ± 184.1	711.9 ± 128.5 (*p* < 0.001*) (*p* < 0.001***)	716.9 ± 101.5	748.9 ± 117.5	809.6 ± 158.4 (*p* < 0.001*) and (*p* < 0.001***)	830.3 ± 158.5	843.0 ± 167.2	875.8 ± 158.2 (*p* < 0.001*, *p* < 0.001**, and *p* < 0.001***)
SDNN [ms]	55.9 ± 27.3	20.9 ± 16.0 (*p* < 0.001*) and (*p* = 0.042***)	22.9 ± 15.5	27.7 ± 18.9	36.7 ± 21.6 (*p* = 0.519*) and (*p* = 0.035***)	38.5 ± 18.1	41.2 ± 20.2	47.1 ± 24.9 (*p* = 1.000*, *p* = 0.005**, and *p* = 1.000***)
RMSSD [ms]	67.5 ± 42.2	17.8 ± 19.8 (*p* < 0.001*) and (*p* = 0.002***)	20.0 ± 19.9	25.5 ± 24.8	37.2 ± 33.1 (*p* = 0.014*) and (*p* = 0.005***)	38.3 ± 29.5	41.7 ± 31.8	50.1 ± 38.0 (*p* = 1.000*, *p* = 0.023**, and *p* = 1.000***)
DFAa1	0.80 ± 0.32	1.23 ± 0.33 (*p* < 0.001*) and (*p* < 0.001***)	1.22 ± 0.29	1.17 ± 0.32	1.06 ± 0.35 (*p* < 0.001*) and (*p* = 0.203***)	1.07 ± 0.33	1.04 ± 0.34	0.98 ± 0.32 (*p* = 0.007*, *p* = 0.067**, and *p* = 1.000***)

Abbreviations: DBP, peripheral diastolic blood pressure; DFAa1, short‐term scaling exponent alpha 1 of detrended fluctuation analysis; HR, heart rate; meanRR, mean RR‐intervals; PWV, pulse wave velocity; RMSSD, root mean square of successive differences of normal‐to‐normal RR‐intervals; SBP, peripheral systolic blood pressure; SDNN, standard deviation of all normal‐to‐normal RR‐intervals.

^*^ compared to PRE.

^**^ compared to POST15.

^***^ compared to MOD.

## 4 Discussion

The aim of the present study was to determine the acute responses of HR time‐series metrics and selected hemodynamic parameters during the recovery phase following two exercise bouts with different intensities. The main focus was to advance the state of research by incorporating the nonlinear metric DFAa1, providing additional qualitative information about ANS regulation and reorganization during recovery.

Internal load analysis of MOD versus VIG revealed significant differences in mean %HR_PEAK_ (∼66% vs. 86%) and %VO_2PEAK_ (∼48% vs. 66%), as well as for blood lactate concentration (∼1 mmol/L vs. 5 mmol/L). These findings support the conclusion that the exercise stimulus was sufficient for the targeted intensity zones corresponding to the “moderate” and “vigorous” domains (Garber et al. 2011; “moderate” to “high” domain within a recent proposal and consensus statement in exercise intensity terminology from Bishop et al. 2025). Our initial hypothesis that the greater homeostatic perturbation induced by the higher exercise intensity results in delayed reorganization and decreased values of linear HRV metrics during recovery could be verified. The present data showed significant main effects of time, intensity, and the interaction for all RR‐interval outcomes, PWV, and SBP. In line with our second hypothesis, the results confirmed the assumption that DFAa1 displayed a stronger correlated reorganization and overcompensation after the more intense exercise bout.

Interpreting, postexercise linear HRV measures (e.g., RMSSD) demonstrate a time‐dependent recovery that is usually, but not always, delayed following a higher preceding exercise intensity (Michael et al. 2017). Exercise intensity appears to be the main driver of immediate postexercise and delayed recovery processes, in addition to factors such as level of performance (Seiler et al. 2007; Altini and Plews 2021). Regarding other prescription variables, such as exercise duration, the isolated influence on postexercise recovery and reactivation processes is not yet fully understood (Michael et al. 2017). This also applies to the interaction of such variables, which alter exercise intensity (e.g., through exercise duration, movement frequency, and also the type and mode of exercise; Hofmann and Tschakert 2017; Gronwald et al. 2020). During early passive postexercise recovery, exercise‐induced sympathetic activation and parasympathetic withdrawal are reversed through the immediate removal of “central command” and decreasing feedback from muscle mechanoreceptors, which also resets the arterial baroreflex to a lower level. This causes an abrupt HR decrease mediated by increased parasympathetic activity as “reactivation” (Kannankeril and Goldberger 2002; Kannankeril et al. 2004; Coote 2010; Peçanha et al. 2014). These changes in autonomic regulation probably reflect compensatory mechanisms for altered hemodynamic processes postexercise, contributing to the recovery of physiological homeostasis (Hautala et al. 2001). Hautala et al. (2001) also described a “rebound phenomenon” in early recovery, where recovery mechanisms are augmented, suggesting a “hypervagal” effect. This pattern could be seen after the VIG condition in the present data of DFAa1. In addition, Buchheit et al. (2007) conducted a study adressing parasympathetic reactivation after repeated sprints and proposed that HRV responses during recovery processes may stem from influences of anabolic metabolism and muscle metaboreflex stimulation. This alters sympathetic activity and blood pressure regulation after exercise termination. In addition, inflammatory responses and immune system activation (e.g., proinflammatory cytokines) could influence ANS regulation patterns and HRV recovery after different exercise intensities (Peake et al. 2017). This could also trigger the production and release of stress hormones (e.g., cortisol), which diminishes the activity of the parasympathetic branch of ANS, potentially delaying the recovery of linear and nonlinear HRV metrics (Tracey 2002).

Regarding the hemodynamic parameters, a significant reduction in PWV, SBP, and DBP was detected during the late recovery period after the VIG condition. This aligns with previous research reporting the postexercise hypotension effect for various exercise protocols (Milatz et al. 2015; Ketelhut et al. 2016). This effect is associated with a reduction in total peripheral resistance, which leads to increased venous pooling and a subsequent drop in central venous pressure and left ventricular preload (Halliwill 2001). This results in a reduction of stroke volume. Although HR increases, the resulting rise in cardiac output is not substantial enough to counteract the drop in total peripheral resistance. As a result, hemodynamic parameters decrease (Kenney and Seals 1993). PWV and SBP also revealed differences between MOD and VIG. This aligns with previous studies showing that higher exercise intensities elicit a more pronounced postexercise hypotension effect (Kircher et al. 2022; Pescatello et al. 2004). Rossow et al. (2010) demonstrated that exercise with higher intensity leads to greater reductions in total peripheral resistance. This is associated with an increased oxygen demand in the working muscles, which provokes greater blood flow through the vessels and thereby enhances shear‐stress‐induced nitric oxide release (Wisløff et al. 2007). In the present study, this effect may have been further amplified by the intermittent nature of VIG exercise, which leads to fluctuations in cardiac output. These fluctuations are considered a driving force for pulsatile shear stress, resulting in a more pronounced release of nitric oxide (Stuckey et al. 2012; Ketelhut et al. 2022, 2023). In a systematic review and meta‐analysis from Price et al. (2020), it could be determined that cardiovascular responses following interval exercise compared to continuous exercise are not substantially different. However, the authors stated that the available evidence is limited and of low quality. In the present data, it could be shown that both modes of exercise with different exercise intensities led to varied characteristics of the internal load situation, leading to perturbation of homeostasis after completion of the more intense exercise bout. In that regard, nonlinear analysis of HR time series revealed interesting recovery kinetics and could provide further useful insights for the analysis of postexercise recovery processes.

Overall, the present data suggest that exercise in the vigorous intensity domain transiently delays the recovery of cardiac parasympathetic activity and normalization of correlation properties of HR time series. In particular, for DFAa1 as a bidirectional measure, a change in the RR interval dynamics was observed, shifting from fractal‐like behavior with DFAa1 close to 1.0 under resting conditions toward a stronger correlation (> 1.2) during early recovery in the VIG condition, with higher values remaining throughout the evaluated recovery process. Under resting conditions, DFAa1 values around 1.0 mirror the homeodynamic behavior of control systems to dynamically self‐organize between order (predictability) and disorder to be able to cope with external and internal demands (Kauffman 1995; A. L. Goldberger et al. 2002; de Godoy 2016). Based on the signal‐theoretical background and the framework of a self‐organized dynamic regulation of the CAN (Benarroch 1993), changes during higher demands are attributed to complex alterations in autonomic modulation. This includes parasympathetic withdrawal, sympathetic activation, altered nonneural factors, and the potential loss of interaction between the two branches of the ANS with increased demands (Persson 1996; White and Raven 2014). In regards to acute early and delayed recovery processes, higher correlation properties could determine more order (less complexity) and interaction of involved control processes, which is reflected by a coherence of fluctuations (e.g., larger fluctuations follow larger fluctuations and vice versa). This leads to the assumption of more systemic control (reorganization and overcompensation) to process the physiological demands of higher exercise intensities within a homeodynamic understanding of the organismic regulation (Hoos and Gronwald 2025).

There are only a few studies investigating DFAa1 during active and passive recovery scenarios (Casties et al. 2006; Platisa and Gal 2008; Mendonca et al. 2010; Blasco‐Lafarga et al. 2013; Boullosa et al. 2014; Gronwald et al. 2019; Martínez‐Navarro et al. 2019). Similarly, these studies also show increased DFAa1 values postexercise across various intensities, durations, and modalities (e.g., incremental test regimes, ultra‐runs, all‐out sport‐specific testing, and high‐intensity training scenarios), implying that other prescription variables and interrelated factors that increase overall physiological demands could lead to similar changes in postexercise reactivation dynamics. However, there is still a lack of sufficient control group comparisons. Far fewer studies have compared different exercise stimuli (e.g., intensity) (Millar et al. 2009; Perkins et al. 2017; Naranjo‐Orellana et al. 2020). Millar et al. (2009) evaluated recovery processing after 1x versus 4x Wingate protocols and could show greater increases in DFAa1 at an earlier recovery stage, which lasted for a longer period during recovery after multiple Wingate testing. These findings are expanded by the present data, highlighting the influence of exercise intensity, when conducting exercise bouts of similar duration. In the study by Perkins et al. (2017), DFAa1 showed no significant deviation from baseline following high‐intensity interval training compared to moderate‐intensity continuous endurance training; although descriptive absolute values were increased immediately postexercise, especially during the more intense workout. In the study by Naranjo‐Orellana et al. (2020), three different exercise intensities were compared with regard to recovery processes and the alteration of DFAa1. A significant increase was observed in the first minutes of recovery, reaching higher values compared to resting for all three conditions. Later, the recovery dynamics differed depending on the intensity level. After low‐intensity exercise in the moderate domain (comparable to MOD in the present study), DFAa1 stabilizes until after 30 minutes of recovery. However, after both higher‐intensity bouts in the heavy and severe domain, DFAa1 continues to increase. Therefore, the present DFAa1 findings are consistent with previous studies and expand the current state of research regarding the influence of different exercise intensity levels on autonomic regulation during passive recovery.

## 5 Limitations

The main advantage of analyzing postexercise recovery processes with DFAa1 is that it allows detecting correlation properties of predominantly nonstationary HR time series data (Peng et al. 1995). However, assessing correlation properties with DFA under variable conditions could be prone to artifact bias, and careful considerations of artifact correction and device comparison should be applied (Rogers et al. 2021). Relating to the study protocol, recovery assessments longer than 45 min postexercise could provide the opportunity to determine when resting values are fully restored. In addition, although there are studies showing the same reactivation pattern related to DFAa1 as in the present study comparing the same mode of exercise (e.g., continuous or intermittent exercise mode, Millar et al. 2009; Naranjo‐Orellana et al. 2020), the different modes of exercise during the MOD and VIG conditions, especially the intermittency could be an influencing factor, potentially leading to alterations of recovery perturbations (Jiménez‐Pavón and Lavie 2017; Jiménez‐Pavón et al. 2019). As current systematic overviews have shown, these unmatched comparisons are common in the scientific literature (Price et al. 2020; Leal‐Menezes et al. 2025), and therefore, it is difficult to conclude which factor contributes mostly to dynamic changes of autonomic regulation during recovery.

In a recent study by Sasso et al. (2024), exercise mode pattern influences the physiologic response to exercise. The participants completed three moderate‐intensity running exercise sessions of equal speed, duration, and total work, with continuous mode, low‐frequency intermittent mode, and high‐frequency intermittent mode. Postexercise data of a low‐intensity submaximal test setting revealed that higher frequencies of modulations can preserve vagal activity and accelerate postexercise recovery (i.e., HR and RMSSD). These patterns suggest intermittent exercise as a potential strategy to alter autonomic impact and acute physiological stress response. The potential mechanisms could contribute to the improved autonomic outcomes associated with intermittent training (Yamamoto et al. 2015; Stöggl and Björklund 2017). Intermittent protocols or training regimes have the potential to enhance the modulation of vagal activity by reducing metabolite accumulation while minimizing muscle mechanoreceptor‐induced vagal withdrawal during phases of passive recovery (Katayama et al. 2018; Peçanha et al. 2021; Sasso et al. 2024).

It is worth noting that other exercise prescription variables, in addition to intensity, could also influence autonomic recovery processes, highlighting the need to address exercise duration as an important independent prescription factor (Hofmann and Tschakert 2017). This also applies to other external load indicators such as movement frequency (e.g., cadence in cycling exercise; Beneke and Leithäuser 2017) or changes in environmental conditions (e.g., heat and hypoxia). To our knowledge, there are no existing studies that address the question of the duration of an, for example.,, low‐intensity exercise bout required to provide the same stimulus as higher exercise intensities. Another example would be how we can use demanding changes in the environment to compensate for factors such as the intensity of an exercise bout. Here, the relationship with individual performance and capacity levels, specific adaptations (e.g., level of performance), and various variables of exercise prescription and environmental conditions also appears to be interesting, as they may potentially influence autonomic recovery processes. Further data from linear and nonlinear HR time series analysis in this regard are urgently needed. Another potential limitation is that the 12‐h abstention from intensive exercise may not have ensured complete ANS recovery for all participants, which could have influenced baseline HRV and recovery measurements.

## 6 Perspective

The monitoring of postexercise linear and nonlinear HRV metrics as a tool for fine‐tuning monitoring processes (Boullosa et al. 2023) and HRV‐guided training remain relatively underexplored. However, it holds significant potential for endurance athletes who undertake high volumes, varied exercise intensities, and/or multiple training sessions per day (Stanley et al. 2013; Lundstrom et al. 2023). This approach can complement resting physiological analyses providing a more comprehensive understanding of recovery and autonomic nervous system regulation (Michael et al. 2017; Lundstrom et al. 2023; Gronwald et al. 2024).

Postexercise monitoring could play a valuable role in guiding the structuring of microcycle or daily programming, tailored to individual cardiac reactivation kinetics (Stanley et al. 2013). Additionally, it can provide important information for intensity control and inform decisions on the necessary duration of recovery (e.g., was the intended low‐intensity training session really low‐intensity or did the duration of the exercise session have a modifying influence on the level of intensity). Standardized submaximal testing procedures have been proposed as a potential approach to evaluate recovery states, acute fatigue, and early signs of overreaching during exercise (Lamberts et al. 2011; Hammes et al. 2016; Decroix et al. 2018).

Easily accessible HRV data acquisition with validated chest belt sensors enables both laboratory and in‐field applications, creating opportunities for real‐time feedback through the assessment of exercise and postexercise data with linear and nonlinear metrics of HR time series (Gronwald et al. 2021; Rogers and Gronwald 2022). In this context, a dimensionless, global, and systemic internal load indicator, such as DFAa1, could offer valuable potential for further investigation in postexercise regimes. Recently, reliability analysis of postexercise recovery metrics, such as HR and RR‐interval metrics (e.g., RMSSD), has been added as a potentially valuable supplement to specific submaximal testing scenarios (Lamberts et al. 2024). However, it should be noted that clear standardization procedures are necessary, and longer analysis intervals are required for the potential inclusion of frequency domain (e.g., high frequency band) or nonlinear domain metrics of RR‐intervals, such as DFAa1 (e.g., ≥ 2 min, Shaffer et al. 2020).

## 7 Conclusion

The present study adds to the state of research and shows that exercise in the vigorous intensity domain transiently delays parasympathetic reactivation. A greater homeostatic perturbation induced by the higher exercise intensity results in delayed reorganization and decreased values of linear HRV metrics during passive recovery. The results also confirmed the assumption that DFAa1 displayed a stronger correlated reorganization and overcompensation after the more intense exercise bout. Higher correlation properties may indicate more order and interaction of the involved control processes managing recovery. This suggests a stronger systemic control to process the demands of higher exercise intensities within a homeodynamic understanding of the organismic regulation. Assessing standardized postexercise linear and nonlinear HRV metrics as a monitoring tool could be a valuable addition for endurance athletes, aiding in the evaluation of regular training sessions, and complementing resting analysis. Further research is needed to verify its potential in guiding the structuring of microcycle and daily programming tailored to individual cardiac reactivation kinetics.

## Author Contributions

S.K. designed the research. S.K., M.M., E.K. and L.R. conducted the experiments and data processing. T.G. conducted data analysis and interpretation. T.G. drafted the manuscript. All authors provided critical comments on the manuscript, read, and approved the final version of the manuscript.

## Funding

None of the authors received funding for this work from any organization, other than salary support for the authors from their respective institutions. The publication was funded by the open access budget of MSH Medical School Hamburg.

## Ethics Statement

The studies involving human participants were reviewed and approved by Institutional Re‐search Committee of the Medical Faculty of the Martin‐Luther‐University Halle‐Wittenberg (reference no.: 2019–177). The participants provided their written informed consent to participate in this study.

## Conflicts of Interest

The authors declare no conflicts of interest.

## Data Availability

The analyzed raw data supporting the conclusions of this article will be made available by the authors without undue reservation.
